# Integrating machine learning with SHAP to uncover multi-tissue molecular signatures in Osteoarthritis progression

**DOI:** 10.1371/journal.pone.0343226

**Published:** 2026-03-09

**Authors:** Jifeng Zhao, Jiasheng Tao, Yizhe Song, Jiyong Yang, Xiaodong Lin, Zhilong Ye, Chao Lu, Mingzhu Zeng, Weijian Chen, Wengang Liu

**Affiliations:** 1 The Fifth Clinical Medical College, Guangzhou University of Chinese Medicine, Guangzhou, Guangdong, China; 2 Guangdong Provincial Second Hospital of Traditional Chinese Medicine (Guangdong Provincial Engineering Technology Research Institute of Traditional Chinese Medicine), Guangzhou, Guangdong, China; 3 Guangdong Provincial Key Laboratory of Research and Development in Traditional Chinese Medicine, Guangzhou, Guangdong, China; University of Vermont College of Medicine, UNITED STATES OF AMERICA

## Abstract

Osteoarthritis (OA) is a chronic joint disorder characterized by pain, reduced mobility, and structural degeneration. Despite its complex etiology and multi-tissue involvement, the molecular mechanisms underlying OA remain poorly understood. This study aimed to identify tissue-specific diagnostic biomarkers using an integrative framework combining multiple machine learning (ML) algorithms and SHapley Additive exPlanations (SHAP). Gene expression profiles from cartilage, synovium, and peripheral blood were retrieved from the GEO database. DEGs were identified across tissues, followed by feature selection using Least Absolute Shrinkage and Selection Operator(LASSO), Support Vector Machine Recursive Feature Elimination (SVM-RFE), and Random Forest(RF). Functional enrichment, gene set variation analysis (GSVA), and immune infiltration analyses were conducted. 10 ML models were constructed to evaluate diagnostic performance. A total of 8, 28, and 61 DEGs were identified in cartilage, synovium, and blood, respectively. Enrichment analysis revealed the key roles in inflammatory signaling, metabolism, and immune pathways. Biomarkers identified included CSN1S1, ABCA6, RARRES1, NPTX2 (cartilage); SCRG1, CXCL2, PTGDS, CCL19, BGN, KLF9 (synovium); and GNL3L, C6orf111, NT5C3, ZNF148 (blood). Immune analysis indicated shifts in mast cells and CD8 + T cells in cartilage and dendritic cells in synovium, while no significant immune alterations were found in blood. Diagnostic models demonstrated strong performance, with AUCs of 0.839 (cartilage), 0.934 (synovium), and 0.892 (blood). SHAP analysis was employed to interpret each model by quantifying the contribution of individual genes to predict outcomes. In the optimal cartilage model, CSN1S1 and ABCA6 were the most influential features, with mean absolute SHAP values of 0.146 and 0.122, respectively. For synovium, SCRG1 (0.111) and CXCL2 (0.097) were top contributors, while in blood, GNL3L (0.148) and C6orf111 (0.143) showed the highest predictive importance. These results underscore the interpretability of the models and validate the functional relevance of selected biomarkers. Collectively, this study provides a robust ML-based framework for identifying and interpreting reliable OA biomarkers across multiple tissues, offering valuable insights into disease mechanisms and supporting the development of diagnostic tools.

## Introduction

Osteoarthritis (OA) is the most common degenerative articular disease, typically affecting one or more joints, and is a leading cause of disability among the elderly [1]. Clinically, it presents with chronic pain, articular stiffness, and loss of mobility [2]. According to the Global Burden of Disease study, approximately 595 million individuals worldwide were affected by OA in 2020 (7.6% of the global population), representing a 132.2% increase since 1990 that likely reflects population growth and aging, a higher prevalence of major risk factors (e.g., obesity and metabolic dysfunction), and improved recognition and diagnosis rather than a confirmed change in OA biology itself [3]. The associated pain, functional impairment, and high medical costs significantly reduce the quality of life in middle-aged and older adults, while also imposing a substantial burden on patients and healthcare systems [4,5]. Unfortunately, existing therapies for OA remain largely palliative, aiming to alleviate pain and manage symptoms, with joint replacement reserved for advanced stages of the disease. These therapeutic strategies have notable limitations. For instance, long-term use of nonsteroidal anti-inflammatory drugs can lead to serious gastrointestinal complications, and the limited lifespan of prosthetic joints often necessitates revision surgeries within a patient’s lifetime [6]. As a result, there has been growing interest in early diagnosis and intervention. However, this remains challenging, as conventional imaging techniques typically detect only advanced stages of the disease, and radiographic findings often fail to reflect the clinical severity of pain [7]. Recent advances in biomarker research offer promising opportunities for early OA diagnosis and for monitoring treatment response.

OA is a whole-joint disease that affects not only cartilage but also subchondral bone, synovium, and surrounding articular tissues, and is closely linked to systemic inflammation [8]. Chondrocytes in OA display a range of phenotypic alterations, including hypertrophy, dedifferentiation, senescence, inflammation, and metabolic dysregulation [1]. Among these, metabolic imbalance and aberrant gene regulation in chondrocytes play key roles in OA pathogenesis [9]. In addition, immune cells in the synovium such as T cells, B cells, macrophages and their secreted inflammatory cytokines (e.g., IL-22, IL-17) exacerbate synovitis and drive disease progression [10]. Recent studies have reported that in patients with end-stage KOA, postoperative pain correlates with disrupted energy metabolism in peripheral blood mononuclear cells (PBMCs) [11]. Furthermore, in vitro experiments demonstrated that modulating reactive oxygen species levels in PBMCs can suppress inflammatory responses in fibroblast-like synoviocytes [12]. Despite advances in OA biomarker research, most studies have focused on a single tissue type, and integrative investigations aimed at identifying biomarkers across multiple tissues remain limited.

**Fig 1:**
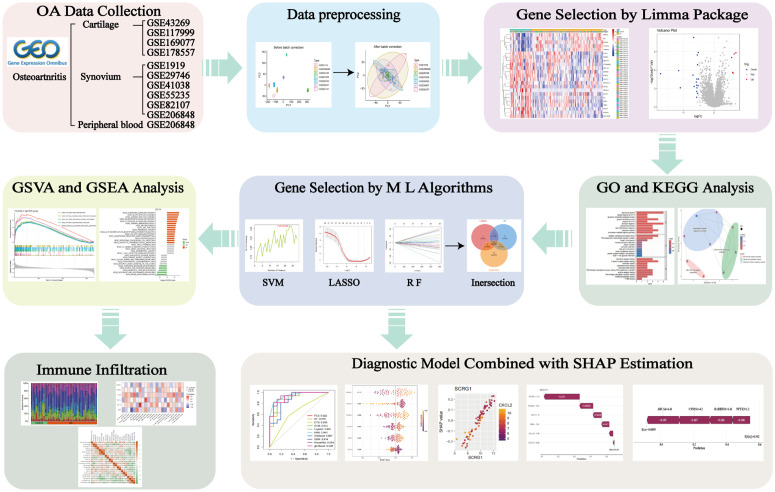
Flowchart of the study design. Transcriptomic data from OA-related tissues, including cartilage, synovium, and peripheral blood samples, were obtained from the GEO database. Differential expression analysis was conducted to identify DEGs. These DEGs were subsequently subjected to GO and KEGG enrichment analyses. Core genes were identified by applying LASSO, SVM-RFE, and RF methods, and overlapping genes among these approaches were selected. GSEA and GSVA were performed to explore the pathways enriched by the core genes. Immune infiltration analysis was conducted to assess the correlation between target genes and immune cell populations. Multiple machine learning models were constructed based on the intersected core genes, and the optimal model was selected using ROC curve analysis. SHAP interpretability analysis was used to elucidate the model’s decision-making process, ultimately identifying genes with diagnostic potential as biomarkers.

As a pivotal tool in data analysis, machine learning algorithms have been widely applied to critical tasks such as disease diagnosis, uncovering potential biological mechanisms, identifying disease-specific patterns, and predicting drug efficacy [13–16]. In particular, machine learning has shown great promise in biomarker identification across various diseases [17]. However, these models often operate as “black boxes,” with complex internal logic that makes it difficult to interpret their decision-making processes and assess their reliability [18]. SHapley Additive exPlanations (SHAP) was developed to address this challenge, providing a robust framework for interpreting machine learning models [19]. By assigning SHAP values to each feature, the method quantifies the contribution of individual variables to model predictions, thereby enhancing interpretability and transparency [20]. In this study, we aim to identify potential biomarkers involved in OA pathogenesis across multiple tissues by mining and analyzing public datasets. By integrating machine learning algorithms with SHAP analysis, we seek to clarify the specific contributions of each key gene (Fig 1), offering a theoretical foundation and molecular basis for early OA diagnosis and the development of targeted therapeutic strategies.

## Materials and methods

### Data collection and preprocessing

Gene expression datasets related to osteoarthritis (OA) were retrieved from the Gene Expression Omnibus (GEO) database (https://www.ncbi.nlm.nih.gov/geo/). The datasets included cartilage samples (GSE43269, GSE117999, GSE169077, and GSE178557), synovial samples (GSE1919, GSE29746, GSE41038, GSE55235, GSE55457, GSE82107, GSE206848), and peripheral blood samples (GSE48556). To eliminate batch effects, the data from each tissue type were independently normalized using the “sva” R package. All datasets used in this study are publicly available (Table 1), and include information on sample size, tissue type, and corresponding annotation platforms. The minimal dataset underlying the findings of this study (including the expression matrix used for machine-learning modeling and SHAP analyses, along with sample group labels) is provided in the Supporting Information (S1 File). All datasets were downloaded from the public GEO database without restriction. Ethical approval and consent to participate are not applicable.

**Table 1:** Statistics of the GEO Datasets.

GEO Accession	Sequencing Method	Sample Tissue	Sample Information		Species	Platform
			OA	Control		
**GSE43269**	RNAseq	Cartilage	23	18	Homo sapiens	GPL8490
**GSE117999**	RNAseq	Cartilage	12	12	Homo sapiens	GPL20844
**GSE169077**	RNAseq	Cartilage	6	5	Homo sapiens	GPL96
**GSE178557**	RNAseq	Cartilage	4	4	Homo sapiens	GPL13497
**GSE1919**	RNAseq	Synovium	5	5	Homo sapiens	GPL91
**GSE29746**	RNAseq	Synovium	11	11	Homo sapiens	GPL4133
**GSE41038**	RNAseq	Synovium	3	4	Homo sapiens	GPL6883
**GSE55235**	RNAseq	Synovium	10	10	Homo sapiens	GPL96
**GSE55457**	RNAseq	Synovium	10	10	Homo sapiens	GPL96
**GSE82107**	RNAseq	Synovium	10	7	Homo sapiens	GPL570
**GSE206848**	RNAseq	Synovium	7	7	Homo sapiens	GPL570
**GSE48556**	RNAseq	Peripheral blood	106	33	Homo sapiens	GPL6947

### Differential expression analysis and functional enrichment analysis

Datasets from the same tissue type were merged, and differential expression analysis was performed using the “limma” R package. Heatmaps and volcano plots were generated using the “ggplot2” and “pheatmap” packages to visualize gene expression differences between OA and normal groups. To further investigate the underlying biological and molecular mechanisms of OA, Gene Ontology (GO) and Kyoto Encyclopedia of Genes and Genomes (KEGG) pathway enrichment analysis were conducted on the differentially expressed genes (DEGs) using the “enrichplot” and “org.Hs.e.g.,db” packages.

### Machine learning-based identification of molecular features

The “glmnet” package was used to perform LASSO regression analysis, which applies penalized minimization of regression coefficients to select genes with significant predictive power from the intersected gene set. The “e1071” package was employed for Support Vector Machine Recursive Feature Elimination(SVM-RFE) analysis, while the “randomForest” package was used for RF analysis. Genes identified concurrently by all three algorithms were considered core genes.

### Single-gene GSEA and GSVA analyses

GSEA and GSVA enrichment analyses were performed using the “clusterProfiler” and “GSVA” packages, with reference gene sets obtained from the Molecular Signatures Database (MSigDB; http://www.gsea-msigdb.org/gsea/msigdb), including “c2.cp.kegg.v7.4.symbols.gmt” for KEGG pathways and “c5.go.v7.4.symbols.gmt” for GO functional annotations. These analyses were conducted to systematically explore the enrichment of biological pathways and functional modules associated with the expression levels of core genes.

### Immune infiltration analysis

To investigate the impact of core genes on immune infiltration in OA, the CIBERSORT deconvolution algorithm was applied to the merged dataset to estimate the relative proportions of 22 immune cell types. The results were visualized using the “ggplot2” package. Spearman correlation analysis was performed to evaluate the relationship between gene expression levels and the relative abundance of immune cells, aiming to elucidate how core genes regulate the immune microenvironment and contribute to OA pathogenesis.

### Construction of OA predictive models

OA predictive models were constructed based on the expression matrix of core genes and optimized using ten different machine learning algorithms, including Partial Least Squares Regression (PLS) [21], RF [22], Decision Tree (DTS) [23], SVM-RFE [24], Logistic Regression [25], K-Nearest Neighbors (KNN) [26], eXtreme Gradient Boosting (XGBoost) [27], Gradient Boosting Machine (GBM) [28], Neural Network [29], and Generalized Linear Model Boosting (glmboost) [30]. To further evaluate the accuracy of these models, the pROC R package was employed to plot receiver operating characteristic (ROC) curves and calculate the area under the curve (AUC), thereby quantifying predictive performance.

### SHAP interpretability analysis

The “kernelshap” package was used to calculate the mean absolute SHAP values of core genes in the optimal model, and the correlations between core genes and their corresponding SHAP values were assessed to evaluate the impact of gene expression levels on prediction outcomes. The “shapviz” package was applied for visualization to determine the contribution of each core gene to the model’s predictions. Finally, force plots and waterfall plots were generated to illustrate the specific contribution of each core gene in the diagnostic decision-making process.

## Results

### Identification of core genes in OA cartilage tissue

**Fig 2:**
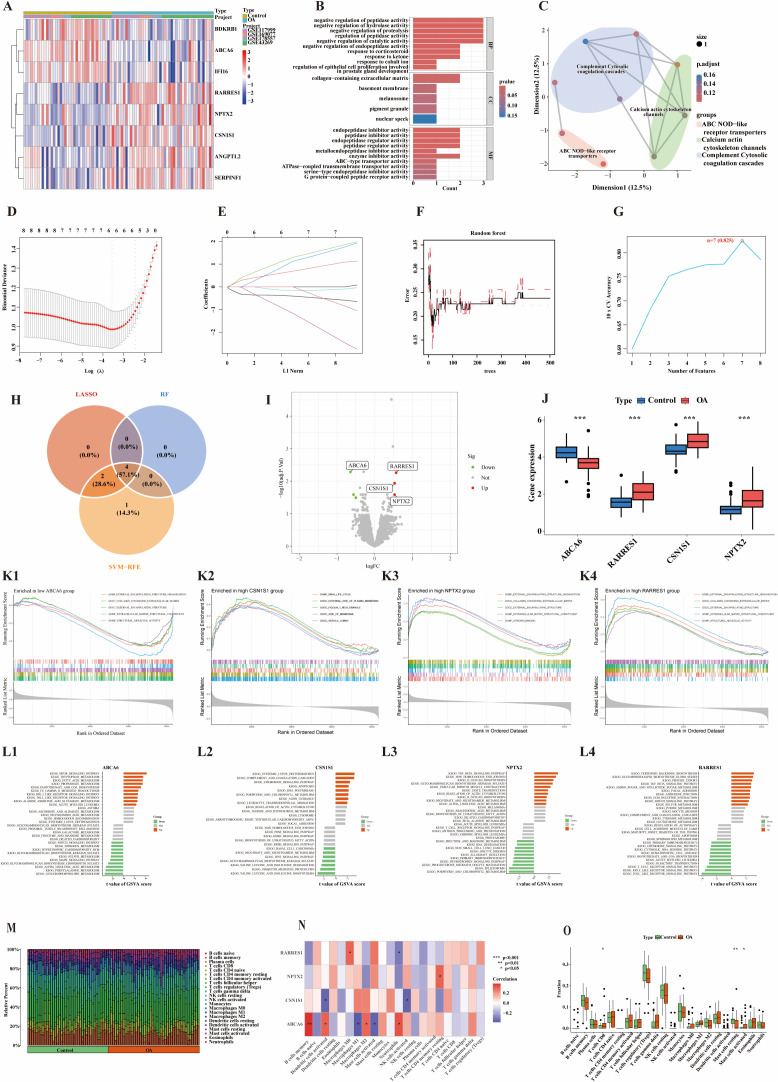
Screening and analysis of core genes in cartilage samples. (A) Heatmap of OA-related DEGs;(B) Bar plot of GO enrichment pathways;(C) Dimensional reduction plot of KEGG enrichment pathways;(D) Binomial deviance presented by the LASSO algorithm;(E) Coefficients presented by the LASSO algorithm;(F) Error rate curve generated by the RF algorithm;(G) Accuracy of the SVM-RFE algorithm;(H) Venn diagram of intersecting genes identified by three machine learning algorithms;(I) Volcano plot of core genes;(J) Box plots of key gene expression levels, *** indicates p < 0.001;(K) GSEA pathway analysis based on GO dataset: K1–ABCA6, K2–CSN1S1, K3–NPTX2, K4–RARRES1;(L) GSVA enrichment bar plots: L1–ABCA6, L2–CSN1S1, L3–NPTX2, L4–RARRES1;(M) Histogram of immune infiltration;(N) Heatmap showing correlations between core genes and immune cells;(O) Box plots comparing immune cell distribution between OA and normal groups.

Cartilage tissue datasets were merged, and batch effects were corrected. Principal component analysis confirmed effective removal of batch effects, and all subsequent analyses were performed using the adjusted principal component scores (S1 Fig A). Differential expression analysis was conducted on cartilage samples using thresholds of |log_2_FC| > 0.5 and adjusted p-value < 0.05, resulting in the identification of eight DEGs, including five upregulated genes (RARRES1, CSN1S1, NPTX2, ANGPTL2, SERPINF1) and three downregulated genes (ABCA6, IFI16, BDKRB1) (Fig 2A).Enrichment analysis of these DEGs revealed significant involvement in GO terms such as negative regulation of peptidase activity, hydrolase activity, proteolysis, and regulation of peptidase activity (Fig 2B). KEGG pathway analysis showed that the DEGs were enriched in pathways including ABC transporters, the cytosolic DNA-sensing pathway, complement and coagulation cascades, and inflammatory mediator regulation of TRP channels (Fig 2C). Integration of three machine learning algorithms—LASSO (Fig 2D and 2E), SVM-RFE (Fig 2F), and RF—identified four core genes: ABCA6, RARRES1, CSN1S1, and NPTX2 (Fig 2G–I). Among them, ABCA6 was significantly downregulated in OA cartilage compared to normal tissue, while CSN1S1, RARRES1, and NPTX2 were significantly upregulated (Fig 2J).

### Core gene analysis in OA cartilage: GSEA, GSVA, and immune profiling

GSEA and GSVA analyses were conducted on the previously identified core genes in OA cartilage tissue (Fig 2K and 2L; S1 Fig B). GSEA results indicated that low expression of ABCA6 was primarily enriched in pathways such as extracellular matrix (ECM)–receptor interaction, glycerophospholipid metabolism, and phenylalanine metabolism (Fig 2K1; S1 Fig B1). In contrast, single-gene GSVA analysis revealed that high ABCA6 expression was associated with increased activity in the mTOR signaling pathway and tryptophan metabolism, while glycerophospholipid and phenylalanine metabolism pathways were suppressed (Fig 2L1). High expression of CSN1S1 was enriched in ECM–receptor interaction, glycosaminoglycan degradation, and lysosome-related pathways (Fig 2K2; S1 Fig B2). GSVA results showed that samples with elevated CSN1S1 expression exhibited activation of the complement and coagulation cascades, whereas the biosynthesis of valine, leucine, and isoleucine and ubiquitin-mediated proteolysis pathways were downregulated (Fig 2L2). For NPTX2, high expression was associated with enrichment in arachidonic acid metabolism, ECM–receptor interaction, and O-glycan biosynthesis pathways (Fig 2K3; S1 Fig B3). GSVA analysis indicated that the TGF-β signaling pathway and non-homologous end joining were activated in cartilage samples with high NPTX2 expression, while spliceosome-related pathways were inhibited (Fig 2L3). High RARRES1 expression was mainly enriched in ECM–receptor interaction, glycosphingolipid biosynthesis – Globo series, and TGF-β signaling pathways (Fig 2K4; S1 Fig B4). Additionally, GSVA revealed that samples with elevated RARRES1 expression showed upregulation of terpene backbone biosynthesis and glycosphingolipid biosynthesis – erythro series, whereas Toll-like receptor and RIG-I-like receptor signaling pathways were suppressed (Fig 2L4).

This study also conducted immune infiltration analysis, revealing differences in the proportions of 22 immune cell types between normal and OA synovial tissues (Fig 2M), as well as correlations among these immune cell types in cartilage tissue (S1 Fig C). Specifically, ABCA6 expression was positively correlated with memory B cells, dendritic cells, M2 macrophages, and neutrophils, but negatively correlated with M1 macrophages and mast cells. CSN1S1 exhibited negative correlations with dendritic cells and natural killer (NK) cells. NPTX2 was positively associated with resting CD4 + memory T cells, while RARRES1 showed positive correlation with M0 macrophages and negative correlation with neutrophils. These findings suggest that the core genes in cartilage may play roles in modulating immune cell populations (Fig 2N). Furthermore, the study found that OA tissues had significantly higher proportions of CD8 + T cells and activated mast cells (*p* < 0.05), and a significantly lower proportion of resting mast cells (*p* < 0.01) compared to normal tissues (Fig 2O), indicating distinct alterations in the immune microenvironment between OA and healthy cartilage.

### Diagnostic modeling and SHAP-based interpretation in OA cartilage

**Fig 3:**
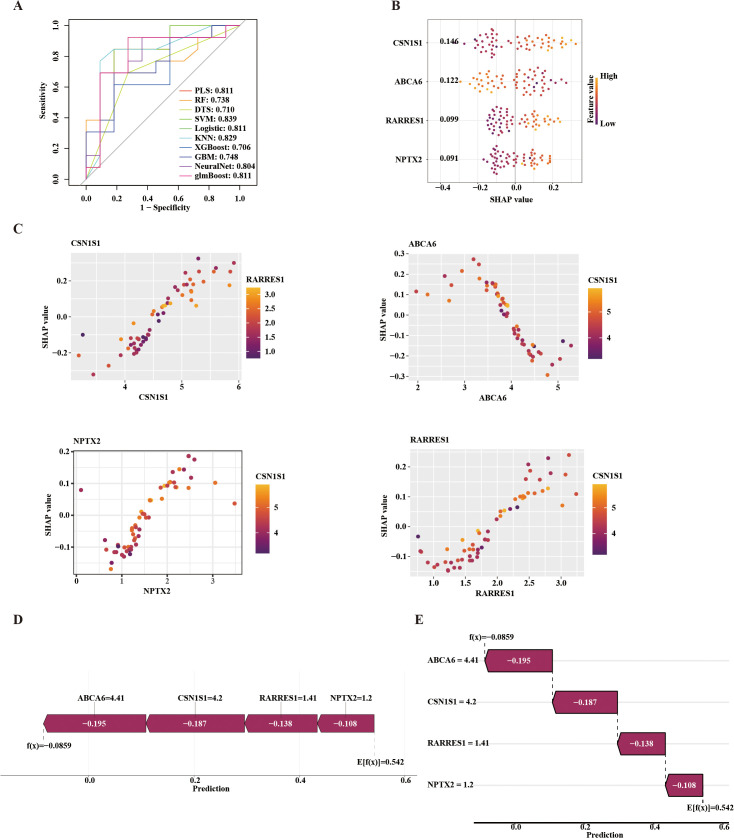
Diagnostic model construction based on core genes in cartilage samples. (A) ROC curves of ten machine learning algorithms;(B) Beeswarm plot showing the relationship between key gene expression and SHAP values;(C) Scatter plot illustrating correlations between key gene expression and SHAP values;(D) SHAP force plot;(E) SHAP waterfall plot.

To further optimize and validate the diagnostic model, multiple machine learning algorithms were employed, including PLS, RF, DTS, SVM-RFE, logistic regression, KNN, XGBoost, GBM, NeuralNet, and glmBoost. The predictive performance of each model was assessed by plotting receiver operating characteristic (ROC) curves and calculating the area under the curve (AUC). Among the models based on cartilage tissue data, the SVM-RFE model achieved the highest performance with an AUC of 0.839 (Fig 3A). To interpret the contribution of core genes to the model’s predictions, SHAP values were calculated for each gene within the SVM-RFE model. CSN1S1 had the highest mean absolute SHAP value (0.146), indicating the strongest influence on the prediction (S1 Fig D). SHAP-based interpretation further revealed that CSN1S1 (0.146), RARRES1 (0.122), and NPTX2 (0.091) were major negative contributors, whereas ABCA6 (0.122) acted as the main positive contributor (Fig 3B). A SHAP scatter plot was generated to examine the relationship between SHAP values and gene expression levels (Fig 3C), showing positive correlations for CSN1S1, RARRES1, and NPTX2, and a negative correlation for ABCA6. To gain a clearer understanding of how core gene expression affects OA prediction, the Shapviz R package was used for interpretability analysis of an individual OA prediction case. A feature contribution force plot (Fig 3D) and a waterfall plot (Fig 3E) were generated. The results showed that ABCA6 (−0.195), CSN1S1 (−0.187), RARRES1 (−0.138), and NPTX2 (−0.108) were ranked in descending order of contribution. Collectively, these genes decreased the model’s prediction from a baseline value of 0.542 to −0.0859, indicating that their expression substantially reduced the predicted OA risk.

### Identification of core genes in OA synovial tissue

**Fig 4:**
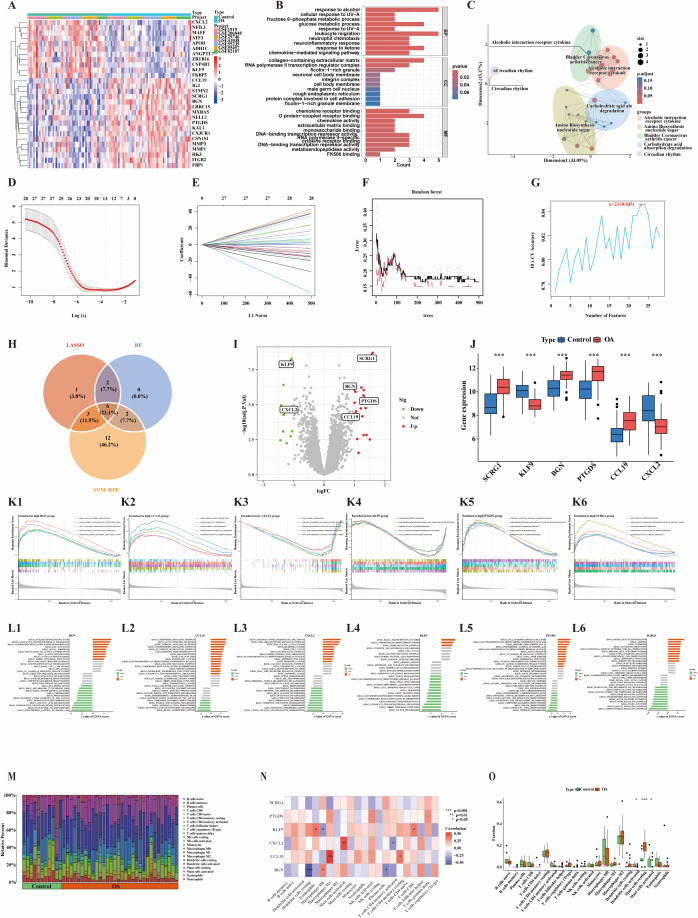
Screening and analysis of core genes in synovial samples. (A) Heatmap of OA-related DEGs;(B) Bar plot of GO enrichment pathways;(C) Dimensionality reduction plot of KEGG pathway enrichment results;(D) Binomial deviance presented by the LASSO algorithm;(E) Coefficients presented by the LASSO algorithm;(F) Error rate curve of the RF algorithm;(G) Accuracy of the SVM-RFE algorithm;(H) Venn diagram of intersecting genes identified by the three machine learning algorithms;(I) Volcano plot of core genes;(J) Box plots of key gene expression levels, *** indicates p < 0.001;(K) GSEA pathway analysis based on GO datasets: K1–BGN, K2–CCL19, K3–CXCL2, K4–KLF9, K5–PTGDS, K6–SCRG1;(L) GSVA enrichment bar plots: L1–BGN, L2–CCL19, L3–CXCL2, L4–KLF9, L5–PTGDS, L6–SCRG1;(M) Histogram of immune cell infiltration;(N) Correlation heatmap between core genes and immune cells;(O) Box plots comparing immune cell distributions between OA and normal groups.

Synovial tissue datasets were merged, and batch effects were corrected. Principal component analysis confirmed effective control of batch effects, and subsequent analyses were performed using the adjusted principal component scores (S2 Fig A). Differential expression analysis of synovial tissue was conducted using thresholds of |log_2_FC| > 1 and adjusted p-value < 0.05, resulting in the identification of 28 DEGs, including 17 upregulated genes (SCRG1, MMP1, IGJ, LRRC15, KAL1, MMP3, PTGDS, NELL1, CX3CR1, ITGB2, MXRA5, CSN1S1, STMN2, CCL19, HK3, BGN, FBP1) and 11 downregulated genes (ZBTB16, NFIL3, KLF9, ADH1C, ATF3, CYP4B1, MAFF, CXCL2, APOD, FKBP5, ANGPTL7) (Fig 4A). GO enrichment analysis showed that these DEGs were primarily involved in biological processes such as glucose metabolism, leukocyte migration, and chemokine-mediated signaling pathways (Fig 4B). KEGG pathway analysis further indicated that these genes were significantly enriched in pathways associated with rheumatoid arthritis, glycolysis/gluconeogenesis, and IL-17 signaling (Fig 4C). Subsequent integration of three machine learning algorithms—LASSO (Fig 4D and 4E), SVM-RFE (Fig 4F), and RF (Fig 4G)—led to the identification of six core genes: SCRG1, KLF9, BGN, PTGDS, CCL19, and CXCL2 (Fig 4H and 4I). Among these, KLF9 and CXCL2 were downregulated in OA synovial tissue compared to normal samples, while SCRG1, BGN, PTGDS, and CCL19 were upregulated (Fig 4J).

### Core gene analysis in OA synovium: GSEA, GSVA, and immune profiling

GSEA and GSVA analyses were conducted on the previously identified core genes in OA synovial tissue (Fig 4K and 4L; S2 Fig B). GSEA results showed that high BGN expression was primarily enriched in pathways such as extracellular matrix (ECM)–receptor interaction and regulation of the actin cytoskeleton (Fig 4K1; S2 Fig B1). Single-gene GSVA analysis further revealed enhanced activity of ECM–receptor interaction pathways in BGN-high synovial tissues, while pathways like neuroactive ligand–receptor interaction were suppressed (Fig 4L1). High CCL19 expression was associated with enrichment in pathways including cell adhesion molecules (CAMs), chemokine signaling, and cytokine–cytokine receptor interactions (Fig 4K2; S2 Fig B2). GSVA results showed upregulation of chemokine signaling in CCL19-high tissues, while the renin–angiotensin system and niacin and nicotinamide metabolism pathways were downregulated (Fig 4L2). Low CXCL2 expression was mainly enriched in pathways related to aerobic respiration, ATP synthesis–coupled electron transport, the respiratory electron transport chain, oxidative phosphorylation, and degradation of branched-chain amino acids (valine, leucine, and isoleucine) (Fig 4K3; S2 Fig B3). GSVA analysis revealed that in CXCL2-high tissues, the NOD-like receptor signaling pathway was upregulated, whereas oxidative phosphorylation and branched-chain amino acid degradation were suppressed (Fig 4L3). Low KLF9 expression was enriched in immune-related pathways, including immune effector processes, cell surface receptor signaling involved in immune regulation, regulation of immune responses, ECM collagen structures, and chemokine signaling (Fig 4K4; S2 Fig B4). GSVA results indicated that in KLF9-high tissues, terpene backbone biosynthesis was upregulated, while antigen processing and presentation pathways were downregulated (Fig 4L4).High PTGDS expression was associated with enrichment in CAMs, chemokine signaling, and cytokine–cytokine receptor interaction pathways (Fig 4K5; S2 Fig B5). GSVA analysis showed that chondroitin sulfate glycosaminoglycan biosynthesis was upregulated in PTGDS-high tissues, while nitrogen metabolism was suppressed (Fig 4L5). High SCRG1 expression was enriched in pathways related to adaptive immune responses, antigen processing and peptide presentation, inflammatory responses, and positive regulation of immune activity (Fig 4K6; S2 Fig B6). Additionally, GSVA analysis revealed increased activity in other glycan degradation pathways in SCRG1-high synovium, whereas adipocytokine signaling was downregulated (Fig 4L6).

Immune infiltration analysis was also performed in this study. The results revealed notable differences in the proportions of 22 immune cell types between normal and OA synovial tissues (Fig 4M), as well as correlations among these immune cells within synovial samples (S2 Fig C). Specifically, KLF9 expression was positively correlated with eosinophils and CD8 + T cells, but negatively correlated with M0 macrophages. CXCL2 showed a positive correlation with activated mast cells and a negative correlation with activated memory CD4 + T cells. CCL19 was positively associated with M1 macrophages and resting memory CD4 + T cells. BGN expression was positively correlated with M0 macrophages, but negatively associated with resting dendritic cells and plasma cells. These findings suggest that core genes in OA synovial tissue may participate in the regulation of immune cell populations (Fig 4N). Moreover, the study found that, compared to normal tissue, OA synovial tissue exhibited a significantly higher proportion of resting mast cells (*p* < 0.001), along with significantly lower proportions of activated dendritic cells and activated mast cells (*p* < 0.05) (Fig 4O). These results underscore distinct alterations in the immune microenvironment between OA and normal synovial tissues.

### Diagnostic modeling and SHAP-based interpretation in OA synovium

**Fig 5:**
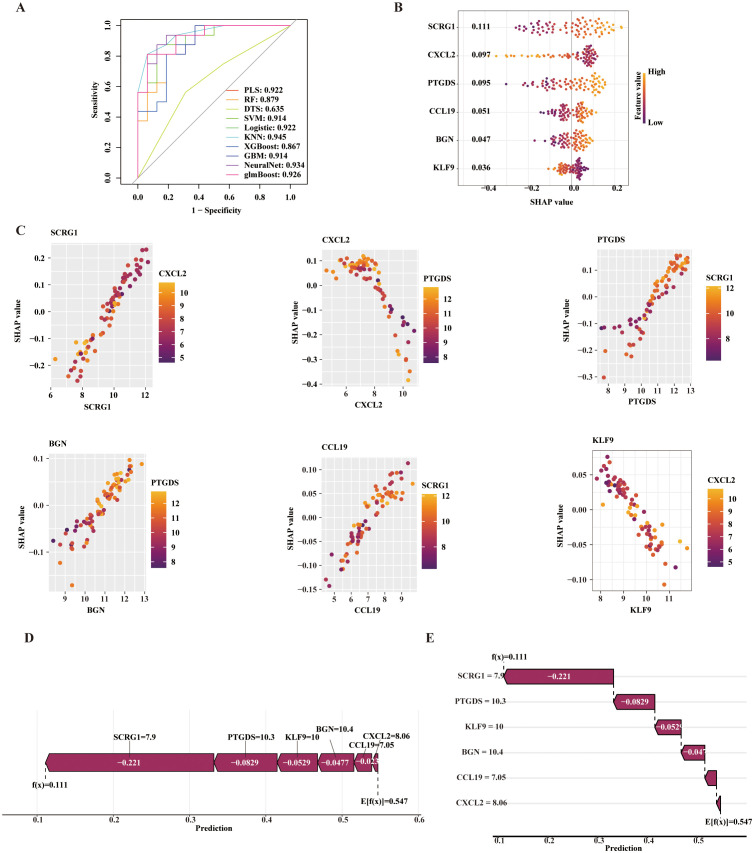
Construction of a diagnostic model based on core genes in synovial samples. (A) ROC curves for ten machine learning algorithms;(B) Beeswarm plot illustrating the relationship between key gene expression and SHAP values;(C) Scatter plots showing correlations between key gene expression and SHAP values;(D) SHAP force plot;(E) SHAP waterfall plot.

Among the diagnostic models constructed using synovial tissue data, the NeuralNet model demonstrated the best performance, achieving an area under the curve (AUC) of 0.934 (Fig 5A). To interpret the contribution of core genes to model predictions, SHAP values were calculated within the NeuralNet model. SCRG1 exhibited the highest mean absolute SHAP value (0.111), indicating the strongest influence on the prediction (S2 Fig D). Furthermore, SHAP-based analysis revealed that SCRG1 (0.111), PTGDS (0.095), CCL19 (0.051), and BGN (0.047) were the primary negative contributors, while CXCL2 (0.097) and KLF9 (0.036) acted as positive contributors (Fig 5B). A SHAP scatter plot (Fig 5C) showed positive correlations between SHAP values and the expression levels of SCRG1, PTGDS, CCL19, and BGN, while CXCL2 and KLF9 were negatively correlated. To further elucidate how core gene expression influences OA risk prediction, a single-case interpretability analysis was conducted using the Shapviz R package. The resulting feature contribution force plot (Fig 5D) and waterfall plot (Fig 5E) indicated that SCRG1 (−0.221), PTGDS (−0.0829), KLF9 (−0.0529), and BGN (−0.0477) were the top contributing genes. Collectively, these genes reduced the model’s predicted risk score from a baseline of 0.547 to 0.111, suggesting that their expression may cooperatively modulate OA susceptibility.

### Identification of core genes in OA peripheral blood samples

**Fig 6:**
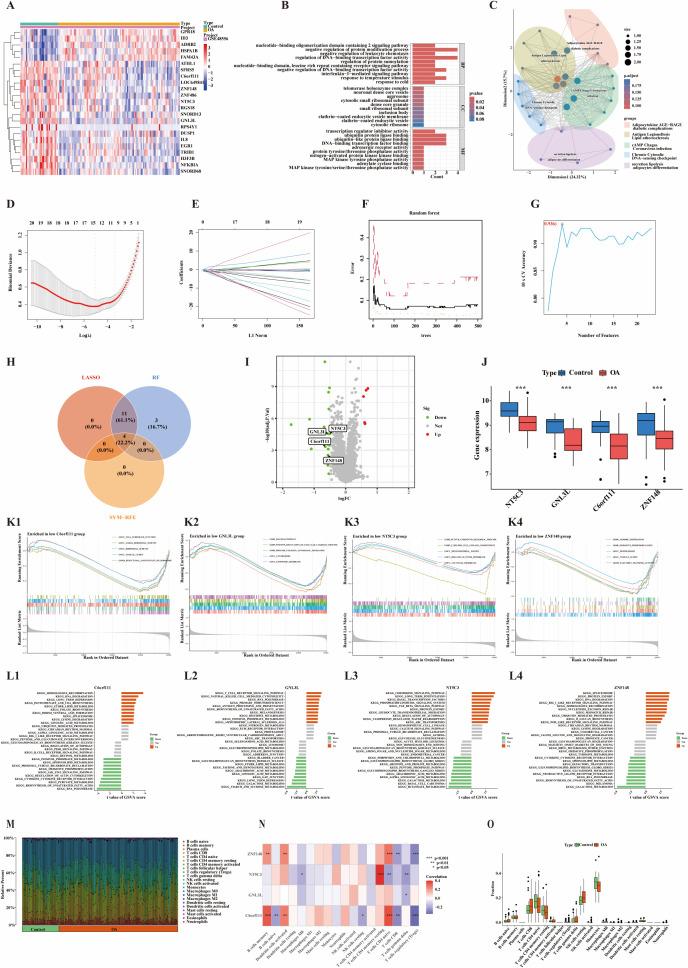
Screening and analysis of core genes in peripheral blood samples. (A) Heatmap of OA-related DEGs;(B) Bar plot of GO enrichment pathways;(C) Dimensionality reduction plot of KEGG enrichment results;(D) Binomial deviance presented by the LASSO algorithm;(E) Coefficients presented by the LASSO algorithm;(F) Error rate curve from the RF algorithm;(G) Accuracy of the SVM-RFE algorithm;(H) Venn diagram showing intersecting genes identified by the three machine learning algorithms;(I) Volcano plot of core genes;(J) Box plots of key gene expression levels, *** indicates p < 0.001;(K) GSEA pathway analysis based on GO datasets: K1–C6orf111, K2–GNL3L, K3–NT5C3, K4–ZNF148;(L) GSVA enrichment bar plots: L1–C6orf111, L2–GNL3L, L3–NT5C3, L4–ZNF148;(M) Histogram of immune cell infiltration;(N) Correlation heatmap between core genes and immune cells;(O) Box plots comparing immune cell distribution between OA and normal individuals.

Since only one dataset of peripheral blood mononuclear cells (PBMCs) was available, batch correction was not performed. Differential expression analysis was conducted using thresholds of |log₂FC| > 0.5 and adjusted p-value < 0.05, resulting in the identification of 23 DEGs, including five upregulated genes (FAM43A, GPR18, ID3, ADRB2, HSPA1B) and eighteen downregulated genes (EGR1, ZNF486, RPS4Y1, C6orf111, GNL3L, NT5C3, etc.) (Fig 6A). Subsequent GO enrichment analysis revealed that these DEGs were primarily involved in biological processes such as negative regulation of protein modification, regulation of DNA-binding transcription factor activity, and negative regulation of DNA-binding transcription factor activity (Fig 6B). KEGG pathway analysis further indicated that these DEGs were significantly enriched in pathways related to Legionellosis, Measles, and Lipid and Atherosclerosis (Fig 6C). Integration of three machine learning algorithms—LASSO (Fig 6D and 6E), SVM-RFE (Fig 6F), and RF (Fig 6G)—resulted in the identification of four core genes: NT5C3, GNL3L, C6orf111, and ZNF148 (Fig 6H and 6I). Compared with normal peripheral blood samples, all four genes were significantly downregulated in OA peripheral blood (Fig 6J).

### Core gene analysis in OA blood: GSEA, GSVA, and immune profiling

Single-gene GSEA and GSVA analyses were performed for the identified core genes in OA peripheral blood samples (Fig 6K and 6L; S3 Fig A). GSEA results indicated that low expression of C6orf111 was primarily enriched in pathways such as antigen processing and presentation, leukocyte transendothelial migration, and oxidative phosphorylation (Fig 6K1; S3 Fig A1). GSVA analysis further revealed that in samples with high C6orf111 expression, pathways including homologous recombination and RNA degradation were activated, whereas the biosynthesis of unsaturated fatty acids was suppressed (Fig 6L1). Low expression of GNL3L was associated with enrichment in pathways related to autophagy, positive regulation of catabolic processes, glycosaminoglycan degradation, lysosome, and NOD-like receptor signaling (Fig 6K2; S3 Fig A2). In contrast, GSVA analysis showed that high GNL3L expression was linked to activation of T cell receptor signaling and NK cell–mediated cytotoxicity, while pathways such as starch and sucrose metabolism and galactose metabolism were downregulated (Fig 6L2). Low expression of NT5C3 was enriched in pathways including amino sugar and nucleotide sugar metabolism, glycosaminoglycan biosynthesis–heparan sulfate, lysine degradation, and the pentose phosphate pathway (Fig 6K3; S3 Fig A3). Meanwhile, GSVA analysis showed upregulation of chemokine signaling in samples with high NT5C3 expression, and suppression of butanoate metabolism (Fig 6L3). Low expression of ZNF148 was primarily enriched in glutathione metabolism, glycolysis/gluconeogenesis, and oxidative phosphorylation (Fig 6K4;S3 Fig A4). GSVA analysis indicated that in samples with high ZNF148 expression, protein export pathways were activated, while galactose metabolism was downregulated (Fig 6L4).

This study also performed immune infiltration analysis, revealing differences in the proportions of 22 immune cell types between normal and OA peripheral blood samples (Fig 6M), as well as correlations among these immune cells within the peripheral blood samples (S3 Fig B). Specifically, NT5C3 expression was positively correlated with resting CD4 memory T cells and negatively correlated with M0 macrophages, naïve CD4 T cells, and γδ T cells. C6orf111 expression was positively associated with memory B cells and activated dendritic cells, but negatively associated with naïve B cells, resting NK cells, CD8 T cells, and regulatory T cells (Tregs). GNL3L showed a negative correlation with γδ T cells. ZNF148 was positively correlated with memory B cells, activated dendritic cells, and naïve CD4 T cells, while showing negative correlations with CD8 T cells and Tregs. These findings suggest that the core genes identified in peripheral blood may play roles in modulating immune cell composition (Fig 6N). However, no significant differences were observed in the overall immune cell landscape between OA patients and healthy controls based on peripheral blood samples (Fig 6O).

### Diagnostic modeling and SHAP-based interpretation in OA blood

**Fig 7:**
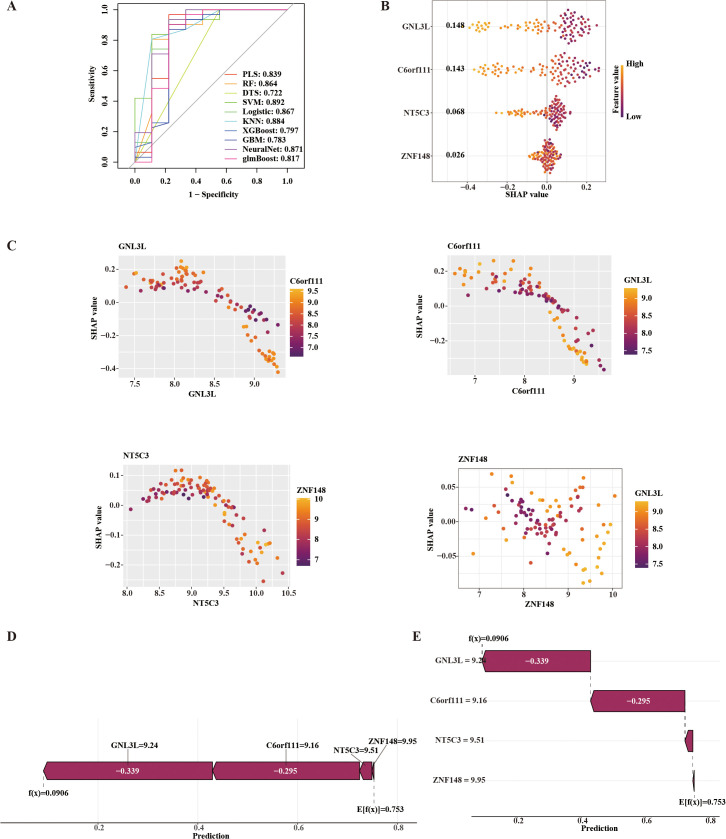
Construction of a diagnostic model based on core genes in peripheral blood samples. (A) ROC curves of ten machine learning algorithms;(B) Beeswarm plot showing the relationship between key gene expression and SHAP values;(C) Scatter plots illustrating the correlations between key gene expression and SHAP values;(D) SHAP force plot;(E) SHAP waterfall plot.

Among the diagnostic models constructed using peripheral blood data, the Support Vector Machine Recursive Feature Elimination (SVM-RFE) model demonstrated the best performance, achieving an area under the curve (AUC) of 0.892 (Fig 7A). To interpret the contribution of core genes to model predictions, SHAP values were subsequently calculated using the SVM-RFE model. GNL3L exhibited the highest mean absolute SHAP value (0.148), indicating the strongest predictive influence (S3 Fig C). SHAP-based interpretability analysis showed that GNL3L (0.148), C6orf111 (0.143), NT5C3 (0.068), and ZNF148 (0.026) all contributed positively to the model’s predictions (Fig 7B). A SHAP scatter plot (Fig 7C) revealed negative correlations between SHAP values and the expression levels of all four genes—GNL3L, C6orf111, NT5C3, and ZNF148. To further elucidate how peripheral blood gene expression influences OA risk prediction, a single-event interpretability analysis was performed using the Shapviz R package. The resulting SHAP force plot (Fig 7D) and waterfall plot (Fig 7E) demonstrated that GNL3L (−0.339) and C6orf111 (−0.295) were among the top contributing genes. Collectively, these genes reduced the model’s predicted risk score from a baseline of 0.753 to 0.0906, suggesting a significant role in decreasing OA susceptibility.

## Discussion

Current clinical diagnostic methods lack precision in assessing the progression of osteoarthritis (OA), and conventional imaging techniques are insufficient for detecting early pathological changes. As a result, biomarker-based approaches for diagnosis and prognostication have become important to OA research. Most existing studies have focused on single-tissue datasets. However, OA is a whole-joint disease that affects the synovium, cartilage, subchondral bone, and peripheral blood. Although some studies have attempted to integrate multi-tissue data through normalization, such strategies may obscure tissue-specific biomarkers uniquely present in distinct compartments. In this study, we applied machine learning and bioinformatics analyses to analyze OA-related gene expression datasets from multiple tissues, aiming to identify tissue-specific diagnostic biomarkers and potential therapeutic targets. We identified core genes in cartilage (ABCA6, RARRES1, CSN1S1, NPTX2), synovium (SCRG1, KLF9, BGN, PTGDS, CCL19, CXCL2), and peripheral blood (NT5C3, GNL3L, C6orf111, ZNF148) that may serve as predictors of OA progression. To assess their diagnostic potential in clinical practice, we constructed tissue-specific OA diagnostic models based on these genes and applied SHAP (SHapley Additive exPlanations) analysis to evaluate the contribution of each biomarker. The results demonstrated robust diagnostic performance across all models, suggesting that these biomarkers may serve as valuable molecular tools for early OA diagnosis, monitoring disease progression, and evaluating therapeutic response.

Among the identified genes, ABCA6 is a cholesterol-responsive gene implicated in lipid metabolism [31]. Studies have shown that ABCA6 contributes to cartilage homeostasis by promoting intracellular cholesterol efflux and delaying chondrocyte senescence [32]. RARRES1 has been reported to promote inflammation by activating the NF-κB signaling pathway [33], which is known to be involved in OA pathogenesis under pathological conditions [34]. We therefore speculate that the link between RARRES1 expression and OA may be mediated through inflammatory mechanisms, although this hypothesis requires further experimental validation. CSN1S1 was found by Brophy et al. to be significantly upregulated in OA meniscus tissue, suggesting it may play an important role in OA, although its precise molecular function remains unclear [35]. In contrast, existing research on NPTX2 has primarily focused on neurological and psychiatric disorders, such as Alzheimer’s disease [36], with limited evidence supporting its involvement in OA.

In addition, downregulation of SCRG1 has been shown to alleviate H₂O₂-induced chondrocyte inflammation, catabolic activity, and autophagy inhibition [37]. Several bioinformatics studies have identified SCRG1 as a potential diagnostic biomarker for OA [38,39]. KLF9 regulates cellular metabolism and function by binding to promoter regions and modulating gene transcription. Overexpression of KLF9 has been reported to exacerbate OA progression by promoting chondrocyte apoptosis and extracellular matrix degradation [40]. The BGN gene encodes a small leucine-rich proteoglycan that undergoes proteolytic cleavage under pathological conditions and is involved in bone formation and mineralization [41]. One study indicated that BGN modulates subchondral bone architecture in traumatic arthritis and cooperates with decorin to regulate cartilage degeneration [42]. A previous transcriptomic bioinformatics analysis confirmed the upregulation of PTGDS in OA synovial tissue [43]; however, its mechanistic role in OA remains to be experimentally validated. CCL19 and its receptor CCR7 are essential components of the adaptive immune system, mediating cell migration and angiogenesis in various pathological contexts [44,45]. Notably, high expression of CCL19 in the synovium has been associated with knee joint dysfunction [46]. CXCL2, a growth-related oncogene, possesses neutrophil chemotactic properties [47]. Bioinformatic studies have suggested that CXCL2, as an apoptosis-associated gene, may serve as a predictive marker for OA onset [48].

Finally, among the four biomarkers identified from peripheral blood, NT5C3 (NT5C3A) encodes an intracellular pyrimidine 5′-nucleotidase involved in the dephosphorylation of nucleoside monophosphates [49]. Although direct evidence linking NT5C3 to OA remains limited, NT5C3A has been implicated in hereditary pyrimidine 5′-nucleotidase deficiency associated with hemolytic anemia, suggesting that blood-based NT5C3 signals may not be OA-specific [50]. GNL3L has been reported to be downregulated in IL-1β–induced OA cartilage and may represent a potential OA-related marker; however, GNL3L has also been reported to be upregulated in acute myeloid leukemia and associated with adverse outcomes, underscoring that circulating transcripts may not be disease-specific [51,52]. C6orf111 (also known as PNISR) is expressed in hematopoietic cells and remains poorly characterized in OA; notably, PNISR has been proposed as a potential diagnostic biomarker in pulmonary arterial hypertension based on transcriptomic analyses with experimental validation [53,54]. ZNF148 is regulated by IL-1β and may modulate inflammatory responses via NF-κB signaling to influence MMP3 expression; it has also been reported to be overexpressed in early-stage colorectal cancer, further suggesting potential overlap with other pathological conditions [55–57]. Collectively, these observations suggest that blood-derived candidates may capture systemic processes that are not necessarily OA-specific; therefore, establishing OA specificity is essential before clinical translation. Accordingly, future studies should validate these candidates in independent, well-phenotyped cohorts that include appropriate disease controls (e.g., other inflammatory arthritides and cardiometabolic comorbidities), apply covariate adjustment (age, sex, BMI, medication use, and systemic inflammatory markers), and evaluate multi-marker panels using stage-stratified and longitudinal designs to improve specificity and clinical interpretability.

Immune cells play a critical role in the onset and progression of OA, primarily by mediating autoimmune responses and secreting chemotactic factors and pro-inflammatory cytokines that disrupt immune homeostasis in periarticular tissues, thereby accelerating OA development [58]. In this study, we observed elevated proportions of CD8 ⁺ T cells and activated mast cells in OA cartilage tissue, alongside a reduction in resting mast cells. In contrast, OA synovial tissue exhibited an increased proportion of resting mast cells, but reduced levels of activated dendritic cells and activated mast cells. Previous studies examining CD8 ⁺ T cell subsets in OA patients at different stages reported a marked increase in pro-inflammatory CD8 ⁺ T cells in the synovial fluid of patients with end-stage OA [59]. Wang et al. demonstrated that reduced mast cell numbers alleviated OA progression in mice, further supporting the pivotal role of mast cell activation in OA pathogenesis [60]. Additional studies have also revealed significant differences in the proportions of resting mast cells, dendritic cells, and M2 macrophages between OA and normal synovial tissues [61],which are largely consistent with our findings.

Finally, this study employed an integrative approach combining SVM-RFE, LASSO regression, and RF algorithms to identify biomarkers across different tissues. In addition, the SHAP interpretability model was applied to evaluate the contribution of each predictive factor to the model’s decisions, thereby confirming the reliability of the constructed diagnostic models. Unlike conventional studies that merge multi-tissue data, our approach emphasizes the diagnostic value of cartilage, synovium, and peripheral blood as independent predictive sources for OA. However, this study primarily relied on publicly available GEO transcriptomic datasets, which may introduce heterogeneity due to differences in sample collection, processing procedures, and microarray platforms. Moreover, many GEO cohorts predominantly include patients with established or late-stage OA, which may bias the identified signatures toward disease-state markers and limit inference regarding molecular alterations in early OA pathogenesis. In addition, due to the limited availability of OA-related gene expression datasets in public repositories, certain tissue-specific datasets had insufficient sample sizes for robust model construction. Furthermore, constrained by experimental limitations, this study was unable to validate the predicted diagnostic biomarkers at the molecular biology level. Future experimental investigations are warranted to confirm the clinical feasibility and diagnostic utility of the identified biomarkers.

## Conclusions

In this study, machine learning algorithms were integrated to identify diagnostically relevant biomarkers in cartilage, synovium, and peripheral blood tissues. The identified biomarkers included ABCA6, RARRES1, CSN1S1, and NPTX2 in cartilage; SCRG1, KLF9, BGN, PTGDS, CCL19, and CXCL2 in synovium; and NT5C3, GNL3L, C6orf111, and ZNF148 in peripheral blood. Diagnostic models were subsequently constructed and optimized based on these biomarkers, achieving favorable accuracy in diagnosing OA and monitoring its progression. Furthermore, immune cells such as CD8 ⁺ T cells and activated mast cells may play critical roles in the pathogenesis and progression of OA, indicating their potential as therapeutic targets in OA immunomodulation.

## Supporting information

S1 File Minimal dataset underlying the findings of this study. Expression matrix used for model training and SHAP analyses, with accompanying sample group labels and metadata.
(ZIP)

S1 Fig Supplementary analysis of cartilage samples. (A) Principal component analysis plots of cartilage samples before and after batch effect correction;(B) GSEA analysis of key cartilage genes using the KEGG dataset: B1 – ABCA6, B2 – CSN1S1, B3 – NPTX2, B4 – RARRES1;(C) Heatmap showing correlations among immune cell types in cartilage samples;(D) Bar plot of SHAP values for core genes in cartilage samples.
(TIF)

S2 Fig Supplementary analysis of synovial tissue samples. (A) Principal component analysis plots of synovial samples before and after batch effect correction;(B) GSEA analysis of key synovial genes based on the KEGG dataset: B1 – BGN, B2 – CCL19, B3 – CXCL2, B4 – KLF9, B5 – PTGDS, B6 – SCRG1;(C) Heatmap of correlations among immune cell types in synovial samples;(D) Bar plot of SHAP values for core genes in synovial tissue.
(TIF)

S3 Fig Supplementary analysis of peripheral blood samples. (A) GSEA analysis of key peripheral blood genes based on the KEGG dataset: A1 – C6orf111, A2 – GNL3L, A3 – NT5C3, A4 – ZNF148;(B) Heatmap showing correlations among immune cell types in peripheral blood samples;(C) Bar plot of SHAP values for core genes in peripheral blood.
(TIFF)

## References

[1] Martel-Pelletier J, Barr AJ, Cicuttini FM, Conaghan PG, Cooper C, Goldring MB, et al. Osteoarthritis. Nat Rev Dis Primers. 2016;2:16072. doi: 10.1038/nrdp.2016.72 27734845

[2] Barnett R. Osteoarthritis. Lancet. 2018;391(10134):1985. doi: 10.1016/s0140-6736(18)31064-x 29864015

[3] GBD 2021 Osteoarthritis Collaborators. Global, regional, and national burden of osteoarthritis, 1990-2020 and projections to 2050: a systematic analysis for the Global Burden of Disease Study 2021. Lancet Rheumatol. 2023;5(9):e508–22. doi: 10.1016/S2665-9913(23)00163-7 37675071 PMC10477960

[4] Hunter DJ, Schofield D, Callander E. The individual and socioeconomic impact of osteoarthritis. Nat Rev Rheumatol. 2014;10(7):437–41. doi: 10.1038/nrrheum.2014.44 24662640

[5] Hawker GA, King LK. The Burden of Osteoarthritis in Older Adults. Clin Geriatr Med. 2022;38(2):181–92. doi: 10.1016/j.cger.2021.11.005 35410675

[6] Magni A, Agostoni P, Bonezzi C, Massazza G, Menè P, Savarino V, et al. Management of Osteoarthritis: Expert Opinion on NSAIDs. Pain Ther. 2021;10(2):783–808. doi: 10.1007/s40122-021-00260-1 33876393 PMC8586433

[7] Glyn-Jones S, Palmer AJR, Agricola R, Price AJ, Vincent TL, Weinans H, et al. Osteoarthritis. Lancet. 2015;386(9991):376–87. doi: 10.1016/S0140-6736(14)60802-3 25748615

[8] Abramoff B, Caldera FE. Osteoarthritis: Pathology, Diagnosis, and Treatment Options. Med Clin North Am. 2020;104(2):293–311. doi: 10.1016/j.mcna.2019.10.007 32035570

[9] Motta F, Barone E, Sica A, Selmi C. Inflammaging and Osteoarthritis. Clin Rev Allergy Immunol. 2023;64(2):222–38. doi: 10.1007/s12016-022-08941-1 35716253

[10] Deligne C, Casulli S, Pigenet A, Bougault C, Campillo-Gimenez L, Nourissat G, et al. Differential expression of interleukin-17 and interleukin-22 in inflamed and non-inflamed synovium from osteoarthritis patients. Osteoarthritis Cartilage. 2015;23(11):1843–52. doi: 10.1016/j.joca.2014.12.007 26521730

[11] Tchetina EV, Glemba KE, Markova GA, Glukhova SI, Makarov MA, Lila AM. Metabolic Dysregulation and Its Role in Postoperative Pain among Knee Osteoarthritis Patients. Int J Mol Sci. 2024;25(7):3857. doi: 10.3390/ijms25073857 38612667 PMC11011761

[12] Lee H-R, Yoo S-J, Kim J, Park CK, Kang SW. Reduction of Oxidative Stress in Peripheral Blood Mononuclear Cells Attenuates the Inflammatory Response of Fibroblast-like Synoviocytes in Rheumatoid Arthritis. Int J Mol Sci. 2021;22(22):12411. doi: 10.3390/ijms222212411 34830290 PMC8624216

[13] Xu J, Pan X, Zhang M, Sun K, Li Z, Chen J. Identification and Validation of the Potential Key Biomarkers for Atopic Dermatitis Mitochondrion by Learning Algorithms. J Inflamm Res. 2025;18:4291–306. doi: 10.2147/JIR.S507085 40144539 PMC11937846

[14] Tang X, Zhou Y, Chen Z, Liu C, Wu Z, Zhou Y, et al. Identification of key biomarkers for predicting CAD progression in inflammatory bowel disease via machine-learning and bioinformatics strategies. J Cell Mol Med. 2024;28(6):e18175. doi: 10.1111/jcmm.18175 38451044 PMC10919158

[15] Yin Y, Cui Q, Zhao J, Wu Q, Sun Q, Wang H-Q, et al. Integrated Bioinformatics and Machine Learning Analysis Identify ACADL as a Potent Biomarker of Reactive Mesothelial Cells. Am J Pathol. 2024;194(7):1294–305. doi: 10.1016/j.ajpath.2024.03.013 38657836

[16] Hounye AH, Xiong L, Hou M. Integrated explainable machine learning and multi-omics analysis for survival prediction in cancer with immunotherapy response. Apoptosis. 2025;30(1–2):364–88. doi: 10.1007/s10495-024-02050-4 39633110

[17] Greener JG, Kandathil SM, Moffat L, Jones DT. A guide to machine learning for biologists. Nat Rev Mol Cell Biol. 2022;23(1):40–55. doi: 10.1038/s41580-021-00407-0 34518686

[18] Cabitza F, Rasoini R, Gensini GF. Unintended Consequences of Machine Learning in Medicine. JAMA. 2017;318(6):517–8. doi: 10.1001/jama.2017.7797 28727867

[19] Wang K, Tian J, Zheng C, Yang H, Ren J, Liu Y, et al. Interpretable prediction of 3-year all-cause mortality in patients with heart failure caused by coronary heart disease based on machine learning and SHAP. Comput Biol Med. 2021;137:104813. doi: 10.1016/j.compbiomed.2021.104813 34481185

[20] Wang Z, Gu Y, Huang L, Liu S, Chen Q, Yang Y, et al. Construction of machine learning diagnostic models for cardiovascular pan-disease based on blood routine and biochemical detection data. Cardiovasc Diabetol. 2024;23(1):351. doi: 10.1186/s12933-024-02439-0 39342281 PMC11439295

[21] Liu C, Zhang X, Nguyen TT, Liu J, Wu T, Lee E, et al. Partial least squares regression and principal component analysis: similarity and differences between two popular variable reduction approaches. Gen Psychiatr. 2022;35(1):e100662. doi: 10.1136/gpsych-2021-100662 35146334 PMC8796256

[22] Hu J, Szymczak S. A review on longitudinal data analysis with random forest. Brief Bioinf. 2023;24(2):bbad002. doi: 10.1093/bib/bbad002 36653905 PMC10025446

[23] Zhang G, Gionis A. Regularized impurity reduction: accurate decision trees with complexity guarantees. Data Min Knowl Discov. 2023;37(1):434–75. doi: 10.1007/s10618-022-00884-7 36618773 PMC9813065

[24] Huang S, Cai N, Pacheco PP, Narrandes S, Wang Y, Xu W. Applications of Support Vector Machine (SVM) Learning in Cancer Genomics. Cancer Genomics Proteomics. 2018;15(1):41–51. doi: 10.21873/cgp.20063 29275361 PMC5822181

[25] Wang QQ, Yu SC, Qi X, Hu YH, Zheng WJ, Shi JX, et al. Overview of logistic regression model analysis and application. Zhonghua Yu Fang Yi Xue Za Zhi. 2019;53(9):955–60. doi: 10.3760/cma.j.issn.0253-9624.2019.09.018 31474082

[26] Abu Alfeilat HA, Hassanat ABA, Lasassmeh O, Tarawneh AS, Alhasanat MB, Eyal Salman HS, et al. Effects of Distance Measure Choice on K-Nearest Neighbor Classifier Performance: A Review. Big Data. 2019;7(4):221–48. doi: 10.1089/big.2018.0175 31411491

[27] Zhao Z, Yang W, Zhai Y, Liang Y, Zhao Y. Identify DNA-Binding Proteins Through the Extreme Gradient Boosting Algorithm. Front Genet. 2022;12:821996. doi: 10.3389/fgene.2021.821996 35154264 PMC8837382

[28] Perez BC, Bink MCAM, Svenson KL, Churchill GA, Calus MPL. Prediction performance of linear models and gradient boosting machine on complex phenotypes in outbred mice. G3 (Bethesda). 2022;12(4):jkac039. doi: 10.1093/g3journal/jkac039 35166767 PMC8982369

[29] Kriegeskorte N, Golan T. Neural network models and deep learning. Curr Biol. 2019;29(7):R231–6. doi: 10.1016/j.cub.2019.02.034 30939301

[30] Li W, Zeng L, Yuan S, Shang Y, Zhuang W, Chen Z, et al. Machine learning for the prediction of cognitive impairment in older adults. Front Neurosci. 2023;17:1158141. doi: 10.3389/fnins.2023.1158141 37179565 PMC10172509

[31] He B, Kang S, Chen Z, Liu X, Wang J, Li X, et al. Hypercholesterolemia risk associated Abca6 does not regulate lipoprotein metabolism in mice or hamster. Biochim Biophys Acta Mol Cell Biol Lipids. 2021;1866(11):159006. doi: 10.1016/j.bbalip.2021.159006 34274505

[32] Wang Y, Wu Q, You Y, Jiang W, Fu P, Dai K, et al. ABCA6 Regulates Chondrogenesis and Inhibits Joint Degeneration via Orchestrated Cholesterol Efflux and Cellular Senescence. Adv Sci (Weinh). 2025;12(10):e2410414. doi: 10.1002/advs.202410414 39823538 PMC11904997

[33] Möller-Hackbarth K, Dabaghie D, Charrin E, Zambrano S, Genové G, Li X, et al. Retinoic acid receptor responder1 promotes development of glomerular diseases via the Nuclear Factor-κB signaling pathway. Kidney Int. 2021;100(4):809–23. doi: 10.1016/j.kint.2021.05.036 34147551

[34] Jimi E, Fei H, Nakatomi C. NF-κB Signaling Regulates Physiological and Pathological Chondrogenesis. Int J Mol Sci. 2019;20(24):6275. doi: 10.3390/ijms20246275 31842396 PMC6941088

[35] Brophy RH, Zhang B, Cai L, Wright RW, Sandell LJ, Rai MF. Transcriptome comparison of meniscus from patients with and without osteoarthritis. Osteoarthritis Cartilage. 2018;26(3):422–32. doi: 10.1016/j.joca.2017.12.004 29258882 PMC6007850

[36] Moreno-Rodriguez M, Perez SE, Nadeem M, Malek-Ahmadi M, Mufson EJ. Frontal cortex chitinase and pentraxin neuroinflammatory alterations during the progression of Alzheimer’s disease. J Neuroinflammation. 2020;17(1):58. doi: 10.1186/s12974-020-1723-x 32066474 PMC7025403

[37] Zhang J, Yuan L, Zhang Y, Jin H, Zhao Y, Zeng X, et al. Loss of SCRG1 in chondrocytes inhibits osteoarthritis by promoting autophagy activity in the temporomandibular joint through inhibition of neurokine receptors. J Oral Facial Pain Headache. 2025;39(1):196–203. doi: 10.22514/jofph.2025.020 40129438 PMC11934739

[38] Liu G, He G, Zhang J, Zhang Z, Wang L. Identification of SCRG1 as a Potential Therapeutic Target for Human Synovial Inflammation. Front Immunol. 2022;13:893301. doi: 10.3389/fimmu.2022.893301 35720295 PMC9204521

[39] Wang X, Liu T, Sheng Y, Zhang Y, Qiu C, Li M, et al. Identification and verification of four candidate biomarkers for early diagnosis of osteoarthritis by machine learning. Heliyon. 2024;10(15):e35121. doi: 10.1016/j.heliyon.2024.e35121 39157341 PMC11328075

[40] Zhou X, Jiang P, Tan H, Wang Y, Bai L. KLF9-GRK5-HDAC6 axis aggravates osteoarthritis pathogenesis by promoting chondrocyte extracellular matrix degradation and apoptosis. Commun Biol. 2025;8(1):23. doi: 10.1038/s42003-025-07460-x 39779910 PMC11711658

[41] Adepu S, Ekman S, Leth J, Johansson U, Lindahl A, Skiöldebrand E. Biglycan neo-epitope (BGN262), a novel biomarker for screening early changes in equine osteoarthritic subchondral bone. Osteoarthritis Cartilage. 2022;30(10):1328–36. doi: 10.1016/j.joca.2022.07.005 35870736

[42] Han B, Li Q, Wang C, Chandrasekaran P, Zhou Y, Qin L, et al. Differentiated activities of decorin and biglycan in the progression of post-traumatic osteoarthritis. Osteoarthritis Cartilage. 2021;29(8):1181–92. doi: 10.1016/j.joca.2021.03.019 33915295 PMC8319061

[43] Tu B, Fang R, Zhu Z, Chen G, Peng C, Ning R. Comprehensive analysis of arachidonic acid metabolism-related genes in diagnosis and synovial immune in osteoarthritis: based on bulk and single-cell RNA sequencing data. Inflamm Res. 2023;72(5):955–70. doi: 10.1007/s00011-023-01720-4 36995411

[44] Yamashita M, Iwama N, Date F, Shibata N, Miki H, Yamauchi K, et al. Macrophages participate in lymphangiogenesis in idiopathic diffuse alveolar damage through CCL19-CCR7 signal. Hum Pathol. 2009;40(11):1553–63. doi: 10.1016/j.humpath.2009.03.021 19540558

[45] Qin Y, He LD, Sheng ZJ, Yong MM, Sheng YS, Wei Dong X, et al. Increased CCL19 and CCL21 levels promote fibroblast ossification in ankylosing spondylitis hip ligament tissue. BMC Musculoskelet Disord. 2014;15:316. doi: 10.1186/1471-2474-15-316 25260647 PMC4190335

[46] Nair A, Gan J, Bush-Joseph C, Verma N, Tetreault MW, Saha K, et al. Synovial chemokine expression and relationship with knee symptoms in patients with meniscal tears. Osteoarthritis Cartilage. 2015;23(7):1158–64. doi: 10.1016/j.joca.2015.02.016 25724256 PMC4470781

[47] Zhou C, Gao Y, Ding P, Wu T, Ji G. The role of CXCL family members in different diseases. Cell Death Discov. 2023;9(1):212. doi: 10.1038/s41420-023-01524-9 37393391 PMC10314943

[48] Yu E, Zhang M, Xu G, Liu X, Yan J. Consensus cluster analysis of apoptosis-related genes in patients with osteoarthritis and their correlation with immune cell infiltration. Front Immunol. 2023;14:1202758. doi: 10.3389/fimmu.2023.1202758 37860011 PMC10582959

[49] Aksoy P, Zhu MJ, Kalari KR, Moon I, Pelleymounter LL, Eckloff BW, et al. Cytosolic 5’-nucleotidase III (NT5C3): gene sequence variation and functional genomics. Pharmacogenet Genomics. 2009;19(8):567–76. doi: 10.1097/FPC.0b013e32832c14b8 19623099 PMC2763634

[50] Bogusławska DM, Skulski M, Bartoszewski R, Machnicka B, Heger E, Kuliczkowski K, et al. A rare mutation (p.F149del) of the NT5C3A gene is associated with pyrimidine 5’-nucleotidase deficiency. Cell Mol Biol Lett. 2022;27(1):104. doi: 10.1186/s11658-022-00405-w 36434495 PMC9700897

[51] Li J, Wu Z, Pan Y, Chen Y, Chu J, Cong Y, et al. GNL3L exhibits pro-tumor activities via NF-κB pathway as a poor prognostic factor in acute myeloid leukemia. J Cancer. 2024;15(13):4072–80. doi: 10.7150/jca.95339 38947394 PMC11212074

[52] Li Y, Dong B. Exploring liquid-liquid phase separation-related diagnostic biomarkers in osteoarthritis based on machine learning algorithms and experiment. Immunobiology. 2024;229(5):152825. doi: 10.1016/j.imbio.2024.152825 38997894

[53] Sinclair PB, Sorour A, Martineau M, Harrison CJ, Mitchell WA, O’Neill E, et al. A fluorescence in situ hybridization map of 6q deletions in acute lymphocytic leukemia: identification and analysis of a candidate tumor suppressor gene. Cancer Res. 2004;64(12):4089–98. doi: 10.1158/0008-5472.CAN-03-1871 15205317

[54] Wu W, Chen A, Lin S, Wang Q, Lian G, Luo L, et al. The identification and verification of hub genes associated with pulmonary arterial hypertension using weighted gene co-expression network analysis. BMC Pulm Med. 2022;22(1):474. doi: 10.1186/s12890-022-02275-6 36514015 PMC9746192

[55] Borghaei RC, Gorski G, Seutter S, Chun J, Khaselov N, Scianni S. Zinc-binding protein-89 (ZBP-89) cooperates with NF-κB to regulate expression of matrix metalloproteinases (MMPs) in response to inflammatory cytokines. Biochem Biophys Res Commun. 2016;471(4):503–9. doi: 10.1016/j.bbrc.2016.02.045 26891870

[56] Borghaei RC, Chambers M. Expression of transcription factor zinc-binding protein-89 (ZBP-89) is inhibited by inflammatory cytokines. Pathol Lab Med Int. 2009;1:7–12. doi: 10.2147/plmi.s6249 20686646 PMC2913887

[57] Essien BE, Sundaresan S, Ocadiz-Ruiz R, Chavis A, Tsao AC, Tessier AJ, et al. Transcription Factor ZBP-89 Drives a Feedforward Loop of β-Catenin Expression in Colorectal Cancer. Cancer Res. 2016;76(23):6877–87. doi: 10.1158/0008-5472.CAN-15-3150 27758879 PMC5379474

[58] Tang K, Sun L, Chen L, Feng X, Wu J, Guo H, et al. Bioinformatics Analysis and Experimental Validation of Mitochondrial Autophagy Genes in Knee Osteoarthritis. Int J Gen Med. 2024;17:639–50. doi: 10.2147/IJGM.S444847 38414629 PMC10898481

[59] Platzer H, Trauth R, Nees TA, Tripel E, Gantz S, Schiltenwolf M, et al. CD8+ T Cells in OA Knee Joints Are Differentiated into Subsets Depending on OA Stage and Compartment. J Clin Med. 2022;11(10):2814. doi: 10.3390/jcm11102814 35628940 PMC9145354

[60] Wang Q, Lepus CM, Raghu H, Reber LL, Tsai MM, Wong HH, et al. IgE-mediated mast cell activation promotes inflammation and cartilage destruction in osteoarthritis. Elife. 2019;8:e39905. doi: 10.7554/eLife.39905 31084709 PMC6516833

[61] Chen Z, Ma Y, Li X, Deng Z, Zheng M, Zheng Q. The Immune Cell Landscape in Different Anatomical Structures of Knee in Osteoarthritis: A Gene Expression-Based Study. Biomed Res Int. 2020;2020:9647072. doi: 10.1155/2020/9647072 32258161 PMC7106908

